# A comparative study of the views of social studies teachers and social studies teacher candidates in Türkiye regarding speaking anxiety

**DOI:** 10.3389/fpsyg.2026.1771643

**Published:** 2026-04-22

**Authors:** Mehmet Oran, Mehmet Tamer Kaya, Ceren Utkugün

**Affiliations:** Speech Anxiety and Social Studies, Afyon Kocatepe University, Afyonkarahisar, Türkiye

**Keywords:** social studies, social studies teachers, speaking anxiety, teacher candidates, Türkiye

## Abstract

This study aimed to reveal the views of social studies teachers and prospective social studies teachers in Turkey regarding their speaking anxiety. The research utilized a phenomenological design, a qualitative research method. The study group consisted of 10 social studies teachers and 20 prospective social studies teachers from Turkey. Criterion sampling, a type of purposeful sampling, was used to select the study group, and participants were chosen from among social studies teachers and prospective teachers who reported experiencing speaking anxiety based on their own statements. A semi-structured interview form consisting of open-ended questions was used as the data collection tool. The data obtained from the semi- structured interview form were subjected to content analysis. The results showed that social studies teachers focused on difficulty in self-expression and fear of not being able to express themselves, while prospective social studies teachers experienced anxiety in terms of stress, tension, and feelings of inadequacy. Furthermore, both teachers and prospective teachers exhibited behavioral and affective responses such as speech impairment, tension, worry, and feeling under pressure when experiencing anxiety. The study revealed that anxiety leads to difficulties in self-expression, and preparation for the topic emerged as an effective coping mechanism. Based on the findings, it is recommended that practical courses on communication and oral expression skills be included in teacher training programs; that psycho-educational interventions be implemented to address the affective dimension of speech anxiety; and that practices be developed to strengthen prospective teachers’ self-efficacy, and systematically impart planned speaking and topic preparation skills. Future research should examine speech anxiety using mixed-methods, including variables such as gender, length of professional experience, class level, and personality traits.

## 1 Introduction

For human beings to understand one another, communicate effectively, and express themselves eloquently, they must possess a strong command of their language. Language is one of the main elements that distinguishes humans from other living things and enables them to socialize (Gomleksiz and Koc Deniz, 2019). Bayraktar (2006) defines language as a natural and ongoing system of symbols that enable people to communicate and convey feelings and thoughts to one another.

Language is an element that enables people in society to communicate with each other in a healthy way and to transfer their culture to future generations. People usually transfer this information through written works or verbally, that is, by speaking (Sevim and Gedik, 2014). In this context, speaking is among the primary skills in the learning process.

Speaking is the act of expressing wishes, requests, etc., feelings, and thoughts in the form of sounds, using phrases called sentences, between at least two people (Ucok, 2004). Oral communication plays a fundamental role in interpersonal interactions and is regarded as one of the most essential competencies (Gunduz, 2007). Thanks to speaking, people can obtain various information and form thoughts and opinions (Sallabas, 2012). Furthermore, verbal communication enables individuals to present themselves effectively to others and to foster positive social relationships within society (Sevim, 2012). However, it should not be forgotten that even cultured, equipped, and knowledgeable people have serious difficulties when speaking in front of a group. Anxiety is considered a significant factor that adversely impacts individuals’ ability to communicate effectively, hindering them from expressing their thoughts and emotions clearly and accurately (Lule Mert, 2015).

Anxiety is defined as the emotional states such as restlessness, unhappiness, and fear, that occur in the inner world of people when they feel hopeless about their expectations for the future (Karcic and Cetin, 2015). According to the Turkish Language Association (2005), anxiety means worrying thoughts, worries, and sadness.

Anxiety, which is caused by people’s emotions and excitement, occurs in people for different reasons and affects human life (Ozdal and Aral, 2005). Speaking anxiety, which is a type of anxiety that makes it difficult for individuals to share their knowledge and thoughts in society, is explained as the feeling of failure and anxiety felt by the person who will make a presentation or speech in front of the community (Katranci and Kusdemir, 2015). Individuals with speaking anxiety experience emotions such as fear, nervousness, anger, and sadness, which can manifest physically through symptoms such as sweating, rapid heartbeat, and stuttering. This anxiety, triggered by speaking, is commonly observed in situations such as delivering a speech assigned as homework, speaking for exam purposes, or giving presentations. Such experiences highlight the significant impact of speaking anxiety on individuals’ ability to communicate effectively and confidently in educational and social contexts (Demir & Melanlıoglu, 2014).

To eliminate speech anxiety, the first thing that needs to be done is to make students aware of the fears that cause these anxieties. If people realize the negative behaviors that cause speech anxiety, they will prefer to cope with them instead of avoiding them. To eliminate or reduce public speaking anxiety, people can be motivated through training, practiced, and their field and mastery knowledge on the subject can be increased (Iscan and Karagoz, 2016). Public speaking anxiety can negatively impact individuals’ ability to express themselves; therefore, to be an effective speaker, it is important to consciously use gestures and facial expressions during the speech to reduce anxiety and to adapt the topic to the age and education level of the audience (Katranci and Kusdemir, 2015).

Speaking, a fundamental element of communication, is an unavoidable part of daily life and professional contexts, though it can sometimes cause anxiety for individuals (Ayres and Hopf, 1993). This situation becomes even more pronounced for teachers, who are responsible for conveying information and establishing interaction. Given that teaching is largely based on effective verbal communication and public speaking skills, speaking anxiety among teachers and teacher candidates can negatively affect their professional competence and self-confidence (Katranci and Melanlioglu, 2013; Durmus and Bas, 2016). A review of the literature reveals that studies on speaking anxiety are largely limited to prospective teachers or students; research addressing the experiences and perceptions of practicing teachers regarding speaking anxiety is relatively limited. A significant portion of existing studies focus on determining speaking anxiety using quantitative measurement tools and do not adequately reveal how this anxiety is experienced within the context of the continuous verbal interaction required by the teaching profession. Furthermore, it is noteworthy that few studies examine the possible effects of professional experience on speaking anxiety by including social studies teachers and prospective social studies teachers within the same study. This study differs from previous studies by examining how speaking anxiety varies with professional role and experience, and by examining the views of social studies teachers and prospective teachers on speaking anxiety from a comparative perspective; in this respect, it aims to offer original and complementary contributions to teacher education and in-service professional development processes.

## 2 Methodology

### 2.1 Research method

This research was designed using a qualitative research method, specifically the phenomenological design. Qualitative research enables the realistic and holistic determination of perceptions and events in their natural environment (Yildirim and Simsek, 2008). The phenomenological design was used because it is better suited to identifying the meanings that teachers and prospective teachers, who constitute the study group, attribute to their thoughts and how they convey them. Phenomenological studies, which aim to discover the structures that exist in people’s minds regarding any given subject, involve discovering the shared meaning of people’s experiences related to a concept (Christensen et al., 2014; Creswell, 2013).

### 2.2 Participants

In determining the study group, criterion sampling, a type of purposeful sampling method, was used, and participants were selected from social studies teachers and teacher candidates who reported experiencing speaking anxiety based on their own statements. According to Maxwell (2018), purposive sampling selects information-rich situations suitable for in-depth research. Information-rich situations allow researchers to access as much information as possible (Patton, 2018). Conversely, criterion sampling involves studying all situations that meet a predetermined criterion or criteria (Yildirim and Simsek, 2008). In this search, the criterion for selecting the teachers and teacher candidates who constitute the study group was the accessibility of the participants and the consideration of the answers these participants could give to the questions within the scope of the research. The study group, within the scope of the study, consists of 10 social studies teachers working in secondary schools in Turkey and 20 social studies teacher candidates continuing their education at Afyon Kocatepe University.

The demographic characteristics of the social studies teachers and teacher candidates participating in the research are as follows:

Two different study groups were included in the study. These were social studies teachers and social studies teacher candidates. Table 1 shows that 5 (50%) of the teachers included in the study were female and 5 (50%) were male. When the settlements where the social studies teachers worked were examined, five teachers worked in the city center and five in the district. The ages of the participating teachers ranged from 28 to 47 years. The teacher’s years of professional experience ranged from 3 to 26 years.

**Table 1:** Demographic characteristics of the participants.

Participants		Variables	f
Social studies teachers	Gender	Woman	5
		Male	5
	Age	Under 30	1
		Between 30–40 years	6
		Over 40 years	3
	Professional Experience	Under 10 years	4
		Between 10 and 20 years	5
		Over 20 years	1
	Place of Duty	Province	5
		District	5
Social studies teacher candidates	Gender	Woman	12
		Male	8
	Class Level	1	5
		2	5
		3	5
		4	5
	Where the Family Lives	Province	15
		District	5

Table 1 shows that 12 (60%) of the teacher candidates included in the research were female, and 8 (40%) were male. Examination of the class levels of the social studies teacher candidates revealed that five teacher candidates from each class level were included in the research. As for the places where the prospective teachers live, 15 families reside in the city center, while 5 families live in the district center.

### 2.3 Data collection tools

In the data collection phase, a literature review was conducted on the subject. A literature review is a process of gathering information from different sources, carried out to eliminate deficiencies in the literature on a subject (Timmins and McCabe, 2005). A semi-structured interview form, commonly used in qualitative research, was used to collect data. Interview forms ensure the use of all questions prepared for the research problem (Yildirim and Simsek, 2008). During the preparation of the semi-structured interview form, a literature review was conducted in line with the research objective, and open-ended questions were formulated. These questions were submitted to experts in Turkish, social studies education, and measurement and evaluation. Based on feedback from these experts, the questions were revised in terms of number, scope, and comprehensibility, resulting in the final interview form.

### 2.4 Data collection and analysis

Before the research, ethics committee approval was obtained from the Afyon Kocatepe University Social and Human Sciences Scientific Research and Publication Ethics Committee with decision numbered 2025/435 dated 19.11.2025.

The data for this research were collected through face-to-face interviews using a semi-structured interview guide with open-ended questions. The collected data were transferred to a Microsoft Word file and subjected to content analysis. Content analysis involves a detailed examination of the data, not recording the brightness of the data, and making it sensitive to distortions in the details of codes, sub-themes, and themes (Yildirim and Simsek, 2008). Codes are created from the interviews, and themes are determined by identifying the commonalities across the codes. In the final stage of data analysis, the data, codes, and themes are presented in tables with diagrams and supported by direct confirmations. While the abbreviation “E” (educator) was used when giving the opinions of the social studies teachers who participated in the research, the abbreviation “T” (teacher candidate) was used to refer to the opinions of the social studies teacher candidates. In the analysis, the reliability formula by Miles and Huberman (1994) was used [Consensus Percentage = Consensus/(Consensus + Disagreement) x 100]. Thus, the research consensus rate was calculated as 100%, and a consensus was reached. The answers given by the social studies teachers in the research based on the interview questions were grouped into 64 codes. Later, these codes were grouped, reducing this number to 37. Some of these codes include: stress-tension, feeling inadequate, sweating, inability to make eye contact, decreased self-confidence, inability to establish social relationships, doing breathing exercises, etc. The answers given by the social studies teacher candidates in the study to the interview questions form were collected under 76 codes. Later, these codes were grouped, reducing the number to 41. Some of these codes are: Difficulty in expressing oneself, getting excited, speaking quickly, feeling sad, fear of not being understood, going to diction courses, etc.

## 3 Findings

The study separately examined the perspectives of social studies teachers and teacher candidates regarding speaking anxiety, highlighting their similarities and differences. In the findings and discussion section, the themes related to teachers’ speaking anxiety were presented first, followed by those concerning the teacher candidates’ speaking anxiety.

### 3.1 The meaning attributed to speech anxiety

The answers given by the social studies teachers and teacher candidates who participated in the research to the question “What does speaking anxiety mean to you?” are as follows in Table 2

**Table 2:** The meanings given to speaking anxiety by social studies teachers and prospective teachers.

Social studies teachers			Social studies teacher candidates		
Code	Participant	f	Code	Participant	f
Difficulty expressing oneself	E2, E4, E5, E6, E7, E8, E9, E10	8	Difficulty expressing oneself	T2, T3, T4, T5, T6, T9, T10, T11, T12, T14, T15, T18, T20	13
Stress-tension	E2, E3, E5, E8, E9, E10	6	Stress-tension	T1, T2, T3, T4, T5, T6, T7, T8, T9, T10, T12, T13, T14, T15, T16, T17, T18, T19	18
The storm inside	E1	1	Decreased self-confidence	T20	1
Bondage	E3	1			

When the answers given by the social studies teachers participating in the study to the question “What does speaking anxiety mean to you?” were examined, the codes of difficulty in expressing oneself (8), stress-tension (6), internal storm (1), and captivity (1) emerged, respectively. When the answers given by the social studies teacher candidates to the question were examined, the codes of stress-tension (pressure, shyness, excitement, fear, panic, uneasiness, hesitation, shame) (18), difficulty in expressing oneself (poor communication) (13), and decreased self-confidence (1) emerged.

Some direct quotation examples regarding the views given by social studies teachers with the code of difficulty in expressing oneself are as follows: E6: “Speaking anxiety means having difficulty expressing myself for me.” expressed his opinion. E8: “Speaking anxiety is the difficulty a person has in expressing himself/herself in front of the public or in one-to-one communication and the tension this brings.” answered. Some direct quotation examples regarding the views given by social studies teacher candidates with the code of difficulty in expressing oneself are as follows: T11: “… Problems such as not being able to express oneself, not being able to introduce oneself to the other person, not being able to convey one’s thoughts, ideas, feelings may be encountered.” T18: “They cannot express themselves comfortably and cannot convey their thoughts beautifully,” expressed his opinion.

Some direct quotation examples regarding the views given by social studies teachers with the stress-tension code are as follows: E5: “For me, when it comes to speaking anxiety, we can say that it expresses the feeling of embarrassment and lack of self-confidence that you experience when giving a speech in front of a group.” She expressed her opinion in the form of. E9: “It is the stress and anxiety that an individual experiences in front of a group,” answered.

Some direct quotation examples regarding the views given by the social studies teacher candidates with the stress-tension code are as follows: T1: “For me, speaking anxiety means the stress and excitement I experience when speaking in front of a group or with one person…” expressed his opinion. T7: “…I get stressed, and my heart beats faster. I can say that these are speaking anxiety for me,” answered.

An example of a direct quote regarding the views given by social studies teachers with the code of an internal storm is as follows: E1: “For me, speaking anxiety is like a storm breaking out inside me, in a sense…” answered.

An example of a direct quote regarding the views given by social studies teachers on the code of captivity is as follows: E3: “A situation that is frequently encountered in students we teach in the speech anxiety course is that the development of anxiety in a person is like captivity for a person…” answered.

An example of a direct quote regarding the views given by the social studies teacher candidates with the code of decreased self-confidence is as follows: T20: “…This also causes decreased self-confidence,” answered.

The research findings indicate that social studies teachers and teacher candidates experience speech anxiety from different perspectives. While social studies teachers perceive anxiety primarily as difficulties in self-expression and the creation of internal tension within the individual, teacher candidates emphasized stress and tension. This suggests that they interpret freedom anxiety through a lack of communicative competence, while teacher candidates, lacking professional experience, tend to experience anxiety more through affective and reactive responses. Furthermore, metaphorical expressions such as “inner storm” and “captivity” were rarely observed in social studies; among teachers, more direct, self- efficacy-driven responses, such as the experience of exile, were more prominent. This explanatory finding reveals that speech anxiety differs depending on the level of experience in both strategic and affective good.

### 3.2 Causes of speech anxiety

The answers given by the social studies teachers and teacher candidates who participated in the research to the question “Can you explain what causes you anxiety when speaking to a group?” are as follows in Table 3.

**Table 3:** Reasons for speaking anxiety of social studies teachers and prospective teachers.

Social studies teachers			Social studies teacher candidates		
Code	Participant	f	Code	Participant	f
Fear of not being able to express oneself	E1, E2, E3, E4, E6, E7, E9	7	Thought of feeling inadequate self	T4, T6, T8, T14, T16, T17, T19, T20	8
Fear of judgment	E1, E2, E6, E10	3	Fear of judgment	T1, T2, T5, T6, T8, T10, T16, T17	8
Thought of feeling inadequate self	E2, E4, E7	3	Fear of not being able to express oneself	T1, T2, T10, T15	4
Attitudes of the other party	E5, E8	2	To be disgraced (to be ridiculed, to be humiliated)	T7, T12, T14	3
Fear of failure	E1	2	Excitability	T3, T7	2
		1	Attitudes of the other party	T11, T16	2
			The crowd of the community	T13, T15	2
			The thought of not meeting expectations	T3	1
			Disturbing looks from listeners	T9	1
			Fear of making mistakes	T17	1
			Shyness	T18	1

When the responses of the social studies teachers participating in the study to the question “Can you explain what causes you anxiety when speaking to a group?” were examined, the following codes emerged, respectively: fear of not being able to express oneself (7), fear of being the center of attention (3), feeling inadequate (3), attitudes of the other party (2), fear of being judged (2), and fear of failure (1). When the responses of the prospective teachers to the question were examined, the following codes emerged, respectively: feeling inadequate (8), fear of being judged (8), fear of not being able to express oneself (4), being embarrassed (being made fun of, humiliated) (3), excitement (2), attitudes of the other party (2), the crowd of the community (2), the thought of not being able to meet expectations (1), disturbing looks of the audience (1), fear of making mistakes (1), and shyness (1).

Some direct quotation examples regarding the views given by social studies teachers with the code of fear of not being able to express oneself are as follows: E3: “For example, sometimes I can experience the fear of not being able to express myself fully in crowded groups in the seminars we attend…” expressed her opinion. E9: “…When I express myself incorrectly, I immediately think about what will happen and try to explain myself in a way that will ensure that I am understood correctly and in the way I want…” answered. An example of direct quotation regarding the views given by social studies teacher candidates with the code of fear of not being able to express oneself is as follows: T15: “Concerns such as slips of the tongue and incorrect pronunciation during speech cause me to worry,” answered.

Some direct quotation examples regarding the opinions given by social studies teachers with the code of fear of judgment are as follows: E6: “The basis of my anxiety lies in the fear of being misunderstood…” expressed her opinion. E10: “…being the focal point of all the people there makes me anxious,” the person expressed his opinion. Some direct quotation examples regarding the opinions given by social studies teacher candidates with the code of fear of judgment are as follows: T1: “I think people will criticize me or evaluate me negatively.” expressed his opinion. T2: “I think the biggest factor is the fear of how people will evaluate me,” responded.

Some direct quotation examples regarding the views given by social studies teachers with the code of feeling inadequate are as follows: E4: “If I am not prepared enough for the subject before giving a speech in front of an audience, I cannot feel that I have full command of the subject…” expressed his opinion. E7: “…The first is whether what I say will create the desired effect on the audience…” responded. Some direct quotation examples regarding the views given by social studies teacher candidates with the code of feeling inadequate are as follows: T8: “I generally worry about not feeling prepared enough.” expressed his opinion. T14: “I think about whether I have enough knowledge about the subject I am explaining,” responded.

Some examples of direct quotes regarding the opinions given by social studies teachers using the code of the other party’s attitudes are as follows: E5: “The biggest reason for my anxiety when speaking to the crowd is the condescending looks people get and the sarcastic way they talk to each other.” E8 “…Critical or indifferent looks from the listeners affect my anxiety,” responded. Some direct quotation examples regarding the opinions given by social studies teacher candidates with the code of the other party’s attitude are as follows:

T11: “Being afraid of the other party’s reaction,” answered.

Some direct quotation examples regarding the views given by social studies teachers and social studies teacher candidates with the codes of fear of failure, be disgraced, excitement, the crowd of the community, not being able to meet expectations, disturbing looks of the audience, fear of making mistakes and shyness are as follows: E1: “…First of all, fear of failure affects me a lot…” expressed her opinion. T12: “I have speaking anxiety, especially when I am talking to my close friends because of the risk of being made fun of,” responded. T7: “My heart beats fast, and I am very afraid of stuttering.” T13: “When there are people around me, anxiety gives way to confidence,” expressed her opinion. T3: “…I think I will not be able to meet expectations,” expressed her opinion. T9: “…For example, some people may uncomfortably look at me,” responded. T17: “First of all, I can say fear of making mistakes.” T18: “Being an introverted and shy person…” answered.

The research findings indicate that social studies teachers and teacher candidates experience anxiety differently when speaking in front of an audience. Social studies teachers associate anxiety more with the fear of not being able to express themselves and the fear of being the center of attention, whereas teacher candidates emphasize self-efficacy and social-evaluation dimensions, such as feeling inadequate and fear of being judged. In both teachers and candidates, social and cognitive factors such as the attitudes of the audience, fear of failure, humiliation, nervousness, and fear of making mistakes emerged as significant factors triggering anxiety. This suggests that experienced teachers evaluate anxiety more in the context of communicative performance and public control, while teacher candidates, due to their lack of professional experience, perceive anxiety more in terms of affective and self-efficacy. The findings reveal that public speaking anxiety is a multidimensional process in both cognitive and affective dimensions and is directly related to the individual’s level of experience.

### 3.3 Consequences of speech anxiety (physical/behavioral)

The answers given by the social studies teachers and teacher candidates who participated in the research to the question “What kind of physical/behavioral do you exhibit when you experience speaking anxiety?” are as follows in Table 4.

**Table 4:** Results of social studies teachers’ and prospective teachers’ speaking anxiety (physical/behavioral).

Social studies teachers			Social studies teacher candidates		
Code	Participant	f	Code	Participant	f
Speech disorder	E1, E2, E3, E4, E6, E8, E10	7	Speech disorder	T1, T2, T3, T4, T5, T6, T8, T9, T11, T12, T13, T15, T16, T17, T18, T19, T20	17
Facial flushing	E2, E4, E5, E6, E7	5	Facial flushing	T4, T7, T9, T10, T12, T13, T18, T19	8
Shivering	E1, E6, E9, E10	4	Shivering	T1, T2, T4, T7, T10, T19, T20	7
Sweating	E1, E2, E9	3	Sweating	T2, T3, T5, T9, T17	5
Rapid heartbeat	E1, E2	2	Inability to make eye contact	T3, T5, T11, T17, T20	5
Difficulty breathing	E2, E10	2	Difficulty breathing	T3, T4, T6, T8, T16	5
Laughing behavior	E3	1	Rapid heartbeat	T2, T14, T15	3
Inability to focus	E5	1	To talk fast	T1, T13	2
Playing with objects	E7	1	Playing with objects	T5, T8	2
Inability to make eye contact	E1	1	Inability to focus	T6, T11	2
Do not be quiet	E1	1	Do not be shy	T12	1
Do not be shy	E1	1	Forgetfulness	T20	1

When the answers given by the social studies teachers participating in the study to the question “What kind of behaviors do you exhibit when you experience speech anxiety?” were examined, the following codes were revealed, respectively: speech disorder (inability to gather words, repeating words, stuttering, speaking rapidly, slipping the tongue, hesitating while speaking, trembling voice, repeating sentences or words, forgetting words, difficulty speaking) (7), blushing of the face (cheek) (5), trembling (hand or body) (4), sweating (hand or limbs) (3), rapid heartbeat (accelerating heart) (2), difficulty breathing (2), laughing behavior (1), inability to focus (1), playing with objects (1), inability to make eye contact (1), becoming silent (1) and being shy (1). When the answers given by the social studies teacher candidates participating in the study to the question “What kind of behaviors do you exhibit when you experience speech anxiety?” were examined, the following codes were revealed, respectively: speech disorder (17), blushing (8), trembling (7), sweating (5), inability to make eye contact (5), difficulty breathing (5), rapid heartbeat (excitement) (3), rapid speech (2), playing with objects (2), inability to focus (2), being shy (1), and forgetfulness (1).

Some of the direct quotation examples regarding the opinions given by social studies teachers with the speech disorder code are as follows: E6: “…First of all, I start to hesitate. When I try to gather the words, sometimes the words in my mouth get knotted, and this makes me even more nervous,” responded. E8: “In such a situation, I panic. When I panic, my speaking speed increases and I start to repeat some sentences,” answered. Some of the direct quotation examples regarding the opinions given by social studies teacher candidates with the speech disorder code are as follows: T11: “…So the words and sentences I am going to say can get mixed up. I may have problems with my pronunciation.” expressed her opinion. T17: “I stutter when I have speech anxiety,” answered.

Some of the direct quotation examples regarding the opinions given by social studies teachers with the code blushing are as follows: E4: “When I experience anxiety, I may physically blush,” expressed his opinion. E5 responded: “…a feeling of warmth overtakes my body, then my cheeks turn red,” answered. Some of the direct quotation examples regarding the opinions given by social studies teacher candidates with the code blushing are as follows: T13: “If I am giving a speech in places like the podium, my face turns red due to this anxiety.” expressed her opinion. T18: “I also get a slight blush,” responded.

An example of a direct quote regarding the views given by social studies teachers with the sweating code is as follows: E9: “When I experience speech anxiety, I usually show physical symptoms such as sweating, shaking, and difficulty speaking.” expressed his opinion. An example of a direct quote regarding the views given by social studies teacher candidates with the sweating code is as follows: T2: “…For example, my hands start to sweat,” answered.

An example of a direct quote regarding the views given by social studies teachers with the code of rapid heartbeat is as follows: E2: “…First of all, my breathing becomes faster, and this can make it difficult for me to gather words,” responded. An example of a direct quote regarding the views given by social studies teacher candidates with the code of rapid heartbeat is as follows: T14: “When I have speaking anxiety, I panic, and my hands and feet get tangled up. My heart pounds,” answered.

An example of a direct quote regarding the views of social studies teachers with the code of difficulty in breathing is as follows: E10: “…After a while, when I still could not get over my excitement, I felt like I was having difficulty in breathing,” answered. An example of a direct quote from social studies teacher candidates experiencing difficulty breathing is as follows: T16: “I cannot breathe comfortably, I become thirsty,” responded.

An example of a direct quote regarding the views given by social studies teachers with the tremor code is as follows: E6: “…My hands tremble, my voice starts to tremble, and this creates more anxiety,” responded. An example of a direct quote regarding the views given by social studies teacher candidates with the tremor code is as follows: T4: “…my hands or legs tremble,” expressed his opinion. T19: “When I experience speech anxiety, it is reflected in my body as a reaction; my hands tremble,” answered.

An example of a direct quote regarding the opinions given by social studies teachers with the laughing behavior code is as follows: E3: “Anxiety manifests itself as laughing behavior in me,” expressed her opinion.

An example of a direct quote regarding the views of social studies teachers with the code of inability to focus is as follows: E5: “…If my anxiety continues, I can never focus, and I make more mistakes while speaking.” An example of a direct quote regarding the views of social studies teacher candidates with the code of inability to focus is as follows: T6: “When I experience speaking anxiety, my mind usually turns to completely off-topic thoughts,” responded.

An example of a direct quote regarding the views given by social studies teachers on the code of playing with objects is as follows: E7: “…I can resort to small escape behaviors such as constantly moving my hands or playing with an object such as a pencil.” expressed his opinion. An example of a direct quote regarding the views of social studies teacher candidates with the code of playing with objects is as follows: T5: “I play with my hands and hair, or if there is an object in my hand, I try to play with it,” answered.

Direct quotation examples regarding the views given by social studies teachers and teacher candidates with the codes of not being able to make eye contact, becoming silent, speaking quickly, being forgetful and being shy are as follows: E1: “When I have speech anxiety, I often have difficulty making eye contact, my eyes usually drift to the floor or I try to focus on something…” expressed her opinion. T3: “My eyes drift to the floor, I have difficulty making eye contact,” responded. T1: “Sometimes I get very excited, so I speak quickly, and this causes me to make more mistakes,” expressed his opinion. T12: “…I also get shy in such a situation,” answered. T20: “When I have speech anxiety, I experience forgetfulness,” answered.

Research findings indicate that social studies teachers and teacher candidates exhibit physical, cognitive, and behavioral responses when experiencing speech anxiety. Both groups most frequently reported speech impairments (stuttering, difficulty stringing words, hesitations, rapid speech), followed by physical symptoms such as blushing, trembling, sweating, palpitations, and difficulty breathing. Additionally, cognitive-behavioral responses such as inability to make eye contact, inability to focus, silence, playing with objects, and shyness emerged as significant indicators of anxiety. While speech impairment and physical symptoms were more frequent in teacher candidates, physical and minor behavioral manifestations of anxiety appeared more limited in experienced teachers. This suggests that experienced teachers tend to be able to control and manage anxiety more effectively through experience, while teacher candidates experience both physical and performance-related symptoms of anxiety more intensely. The findings reveal that speech anxiety is a multidimensional process with biological, behavioral, and cognitive dimensions, manifesting differently depending on an individual’s level of experience.

### 3.4 Consequences of speaking anxiety (affective, emotion)

The answers given by the social studies teachers and teacher candidates who participated in the research to the question “How do you feel when you have speaking anxiety?” are as follows in Table 5.

**Table 5:** Results of speaking anxiety (affective emotion, etc.) of social studies teachers and prospective teachers.

Social studies teachers			Social studies teacher candidates		
Code	Participant	f	Code	Participant	f
Affective nervous-anxious-stressed-panicky-affective under pressure	E1, E2, E5, E6, E7, E8, E9	7	Affective nervous-anxious-stressed-panicky-affective under pressure	T1, T2, T3, T4, T5, T6, T7, T8, T9, T10, T11, T18, T19, T20	13
Shy-affective shy-anxious	E2, E4, E5, E6	4	Shy-Affective Shy-Anxious	T1, T2, T4, T6, T9, T13, T17, T18, T20	9
Decreased self-confidence	E2, E3, E10	3	Lack of knowledge-Affective inadequate self	T3, T4, T3, T11, T12, T15	6
Lack of knowledge-affective inadequate self	E4, E9	2	Do not feel sad	T14, T16, T17	3
Annoyed	E5, E10	2	Decreased self-confidence	T5, T8	2
Affective of loneliness	E6	1			
Affect of disgrace	E3	1			

When the answers given by the social studies teachers participating in the study to the question “How do you feel when you have speech anxiety?” were examined, the following codes were revealed, respectively: tense-anxious-stressed-panicky-affective under pressure (7), shy-affective ashamed-uneasy (4), decreased self-confidence (3), ignorance-affective inadequate (2), nervousness (2), affective lonely (1) and affective disgraced (1). When the answers given by the social studies teacher candidates participating in the study to the question “How do you feel when you have speech anxiety?” were examined, the following codes were revealed, respectively: tense-anxious-stressed-panicky-affective under pressure (13), shy-affective ashamed-uneasy (9), ignorance-affective inadequate (6), affective sad (3) and decreased self-confidence (2).

Direct quotation examples regarding the views of social studies teachers coded as affective tense-anxious-stressed-panicky-under pressure are as follows: E7: “When I have speaking anxiety, I feel under pressure both physically and emotionally…” expressed his opinion. E8: “When I have speaking anxiety, I feel quite tense and stressed…” responded. Direct quotation examples regarding the views of social studies teacher candidates coded as affective tense-anxious-stressed-panicky-under pressure are as follows: T1: “When I have speaking anxiety, I usually feel very tense.” expressed his opinion. T10: “When I have speaking anxiety, I feel under intense stress, both physically and emotionally,” answered.

Direct quotation examples regarding the views given by social studies teachers with the code of being shy-affective shy-anxious are as follows: E2: “…this makes me even more shy than I am.” expressed his opinion. E4 responded: “When I am experiencing anxiety, I get a little anxious and this anxiety causes me to feel ashamed…” Direct quotation examples regarding the views given by social studies teacher candidates with the code of being hy-affective shy-anxious are as follows: T9: “I feel ashamed and have a feeling of whether I can succeed or not.” answered. T13: “When I have speaking anxiety, I think everyone is looking at me. I get excited,” answered.

An example of a direct quote regarding the views given by social studies teachers with the code of decreased self-confidence is as follows: E10: “…This situation causes my self- confidence to decrease.” An example of a direct quote regarding the views of social studies teacher candidates with the code of decreased self-confidence is as follows: T8: “I feel like my self-confidence is a little shaken at the moment,” answered.

An example of a direct quote regarding the views given by social studies teachers with the code of ignorance-affective inadequate is as follows: E9: “…I not only feel inadequate, but I also feel uninformed about the subject I am speaking about.” Some examples of direct quotes regarding the views of social studies teacher candidates with the code of ignorance, and affective inadequacy are as follows: T11: “I usually feel inadequate in such situations.” expressed her opinion. T12 stated the following: “…I feel very inadequate about this subject,” answered.

Some direct quotation examples regarding the views given by social studies teachers and teacher candidates with the codes of affective anger, loneliness, affective of disgrace, and sadness are as follows: E5: “When I have speaking anxiety, I can get angry with the oppressive questions coming from the other party.” expressed her opinion. E6: “…it makes me feel more lonely and increases my anxiety. I feel like I have been humiliated…” answered. T14: “When I have speaking anxiety, I get sad…” responded.

Research findings indicate that social studies teachers and teacher candidates exhibit intense emotional and affective responses when experiencing speech anxiety. Both groups most frequently reported affective states of tension, stress, anxiety, panic, and being under pressure, followed by shyness, embarrassment, nervousness, and decreased self-confidence. Furthermore, both teachers and candidates experience feelings of inadequacy, ignorance, loneliness, irritability, and humiliation during anxiety episodes. Teacher candidates experience anxiety and shyness more intensely than experienced teachers; this suggests that lack of experience strengthens the affective dimension of anxiety. The findings reveal that speech anxiety is a multidimensional process with physical, behavioral, and affective dimensions, and that the intensity of emotional responses can vary depending on an individual’s level of experience.

### 3.5 Situations and behaviors interfered with by speech anxiety

The answers given by social studies teachers and teacher candidates participating in the study of the question “What situations and behaviors are affected by speech anxiety in you?” are as follows in Table 6.

**Table 6:** The situations and behaviors affected by speech anxiety in social studies teachers and teacher candidates.

Social studies teachers			Social studies teacher candidates		
Code	Participant	f	Code	Participant	f
Difficulty expressing oneself	E1, E2, E3, E4, E5, E6, E7, E10	8	Difficulty expressing oneself	T1, T2, T3, T4, T5, T6, T8, T10, T11, T12, T13, T14, T16, T17, T18	15
Decreased self-confidence	E1, E2, E4, E5, E6, E8, E10	7	Inability to establish social relationships	T1, T2, T4, T5, T6, T7, T8, T10, T12, T14, T15, T19, T20	13
Shyness/timidity	E3, E7	2	Decreased self-confidence	T1, T3, T4, T7, T9	5
Inability to establish social relationships	E1	1	Fear of not being understood	T1, Ö3	2
Stress/anxious	E9	1			

When the responses of the social studies teachers participating in the study to the question “What situations and behaviors does speech anxiety affect you in?” were examined, the following codes emerged, respectively: difficulty in expressing oneself (8), decreased self- confidence (7), shyness/timidity (2), inability to establish social relationships (1) and nervous/anxious (1). When the responses of the social studies teacher candidates participating in the study to the question “What are the situations in which speaking anxiety prevents you?” were examined, the following codes emerged, respectively: difficulty in expressing oneself (15), inability to establish social relationships (13), decreased self-confidence (5) and fear of not being understood (2).

Direct quotation examples regarding the views given by social studies teachers with the code of difficulty in expressing oneself are as follows: E4: “When I experience anxiety, this situation prevents me from expressing myself.” expressed her opinion. E5: “The situation that speech anxiety causes the most obstacle for me is that I cannot express myself very clearly…” responded. Direct quotation examples regarding the views given by social studies teacher candidates with the code of difficulty in expressing themselves are as follows: T8: “Sometimes I feel that I cannot express myself fully.” expressed his opinion. T13: “Speaking anxiety can prevent me from expressing myself clearly or from speaking understandably,” expressed her opinion. T14: “When I have speaking anxiety, I have a hard time expressing myself…” responded.

Direct quotation examples regarding the views given by social studies teachers with the code of decreasing self-confidence are as follows: E2: “My self-confidence in communication decreases…” expressed his opinion. E8: “My self-confidence can decrease for a while, especially after a conversation that I have difficulty with…” responded. Direct quotation examples regarding the views given by social studies teacher candidates with the code of decreasing self-confidence are as follows: T3: “My self-confidence decreases…” expressed her opinion. T7: “When I experience speaking anxiety, my self-confidence decreases a lot,” responded.

Direct quotation examples regarding the views given by social studies teachers with the code of not being able to establish social relationships are as follows: E1: “I withdraw more due to anxiety, which prevents me from establishing social relationships.” expressed her opinion. Direct quotation examples regarding the views given by social studies teacher candidates with the code of not being able to establish social relationships are as follows: T6: “Speech anxiety usually causes me difficulty in matters such as making new friends in my social life.” expressed his opinion. T15: “Speech anxiety sometimes prevents my social relationships from progressing positively,” expressed her opinion. T20 responded: “My social relationships weaken….”

Examples of direct quotes from social studies teachers and teacher candidates regarding their views coded as shyness/timidity, nervous/anxious, and fear of not being understood are as follows: E3: “To be honest, I do not experience this anxiety very often, but in situations where I do, it can be reflected as shyness.” expressed her opinion. E9: “…I feel nervous and anxious in such situations,” expressed his opinion. T1: “Not being understood also increases my anxiety…” responded.

The research findings reveal that speech anxiety significantly impacts the daily and professional functioning of both social studies teachers and teacher candidates. Both groups reported that anxiety most often affects difficulty in self-expression and decreased self- confidence. Furthermore, teacher candidates emphasized that anxiety also creates obstacles in social dimensions, such as fear of forming social relationships and fear of being misunderstood, suggesting that a lack of social and communication experience exacerbates anxiety. Teachers, on the other hand, stated that anxiety manifests more as introversion, shyness, and timidity. These findings demonstrate that speech anxiety plays a decisive role in personal expression, social interaction, and self-confidence.

### 3.6 Ways to prevent speech anxiety

The responses of the social studies teachers and teacher candidates who participated in the study to the question “What experiences have you had that you think were useful in reducing or eliminating your speaking anxiety?” are as follows in Table 7.

**Table 7:** Situations aimed at reducing or eliminating speaking anxiety in social studies teachers.

Social studies teachers			Social studies teacher candidates		
Code	Participant	f	Code	Participant	f
Preparing for the topic	E1, E2, E4, E5, E7, E9, E10	7	Preparing for the topic (rehearsal, practice)	T1, T2, T3, T4, T6, T8, T10, T12, T13, T14, T15, T16, T17, T18, T20	15
			To be breathing exercises	T1, T3, T4, T8, T10, T19	6
To be breathing exercises	E1, E2, E3, E6, E9	5	Go to the diction course	T7, T11	2
			Socializing	T5	1
Accepting that making mistakes is natural	E1, E2, E7	3	Making eye contact with the listener	T9	1
Making eye contact with the listener	E5, E8	2	Stay calm	T11	1

When the responses of the social studies teachers participating in the study to the question “What experiences do you think were useful in reducing or eliminating your speaking anxiety?” were examined, the following codes emerged, respectively: preparing for the topic (7), doing breathing exercises (5), accepting that making mistakes is natural (3), and making eye contact with the audience (2). When the responses of the social studies teacher candidates participating in the study to the question “What experiences do you think were useful in reducing or eliminating your speaking anxiety?” were examined, the following codes emerged, respectively: preparing for the topic (rehearsing, practicing) (15), doing breathing exercises (6), taking a diction course (2), socializing (1), making eye contact with the audience (1), and staying calm (1).

The direct quotation examples related to the opinions of social studies teachers on the code of preparing for the subject are as follows: E1: “…First of all, preparation is very important.” expressed her opinion. E9: “Being well prepared about the subject I will speak on helps me reduce anxiety,” responded. The direct quotation examples related to the opinions of social studies teacher candidates with the code of preparing for the subject are as follows: T2: “Frankly, one of the most effective methods was practicing.” expressed her opinion. T3: “Preparing in advance helped me reduce my speaking anxiety,” she expressed her opinion. T8: “For example, being well prepared for the subject I will speak on makes me incredibly relaxed.” responded.

The direct quotation examples regarding the views given by social studies teachers on the code of doing breathing exercises are as follows: E2: “Practicing deep breathing techniques and calming myself down before speaking was also effective in controlling my anxiety.” expressed his opinion. E4: “…if you have sufficient command of speaking, there will not be an environment of anxiety anyway,” expressed his opinion. The direct quotation examples regarding the views given by social studies teacher candidates with the code of doing breathing exercises are as follows: T1: “Taking deep breaths and trying to calm down works to reduce my speaking anxiety.” expressed his opinion. T19: “From my experience, doing breathing exercises work when I am going to make a speech.” answered.

Examples of direct quotes regarding the views of social studies teachers coded as making eye contact with the listener are as follows: E5: “I think the most important thing is to be able to make eye contact with the other person…” expressed her opinion. Examples of direct quotes regarding the views of social studies teacher candidates coded as making eye contact with the listener are as follows: T9: “Making eye contact with my close friend to reduce this anxiety calmed me down and minimized my speaking anxiety.”

Examples of direct quotes from social studies teachers regarding the codes of accepting that making mistakes is natural, taking a diction course, socializing, and staying calm are as follows: E7: “Accepting that making mistakes is natural and not being too hard on myself also helped in this process.” expressed his opinion. T7: “I enrolled in a diction course.” expressed his opinion. T5: “Socializing with people reduces this anxiety,” expressed her opinion. T11: “I try to stay calm and not rush about what I have to say,” answered.

Research findings indicate that social studies teachers and teacher candidates believe that preparation and rehearsal are the most effective methods for reducing speaking anxiety. Both groups emphasized that mastering the topic before speaking reduced anxiety. Furthermore, both teachers and candidates reported trying to manage physical symptoms with breathing exercises and calming techniques, while some participants stated that making eye contact with the audience provided confidence and relaxation. Teachers also stressed that they could manage anxiety by accepting that making mistakes is natural; candidates, on the other hand, tried to reduce speaking anxiety with additional strategies such as diction courses and socialization. These findings reveal that both cognitive preparation and physical and social strategies are effective in coping with anxiety.

## 4 Conclusion, discussion, and recommendations

This study aimed to compare the views of social studies teachers and teacher candidates in Türkiye regarding speaking anxiety. The study examined the views of social studies teachers and teacher candidates based on the following themes: the meaning they attribute to speaking anxiety, the causes of speaking anxiety, the consequences of speaking anxiety (behavioral), the consequences of speaking anxiety (emotions, feelings), situations and behaviors interfered with by speech anxiety, and their experiences in preventing speaking anxiety.

When comparing the participants’ views on the meaning they attribute to speaking anxiety, it was determined that social studies teachers focused more on the difficulty of self- expression, while teacher candidates perceived speaking anxiety in terms of stress and tension. This indicates that teachers, based on their professional experience, associate speaking anxiety more with communicative competencies, while teacher candidates, due to their lack of professional experience, evaluate the speaking process more intensely through emotional responses.

When comparing the participants’ views on the meanings they attribute to the causes of speaking anxiety, it was concluded that social studies teachers mostly associated this anxiety with the fear of not being able to express themselves, while social studies teacher candidates based speaking anxiety on feelings of inadequacy. This situation indicates that, depending on their professional experience, they tend to evaluate the speaking process more in terms of performance and communication competence, while prospective teachers, due to their limited experience, focus their anxiety on their self-efficacy perceptions.

When comparing physical/behavioral exhibited while experiencing speech anxiety, both social studies teachers and social studies teacher candidates focused on speech disorders. This indicates that speech anxiety manifests with similar physical/behavioral responses regardless of professional experience level, and that anxiety is a common problem area that negatively affects speech fluency.

When comparing emotions, affectives, and other mental processes that arise while experiencing speech anxiety, both social studies teachers and social studies teacher candidates focused on affectives of tension, worry, stress, panic, and pressure. This shows that speech anxiety has similarly strong effects on emotional and mental levels in both experienced teachers and teacher candidates, and that the negative effects of anxiety on performance are a common problem.

A comparison of the situations and behaviors affected by speech anxiety revealed that both experienced social studies teachers and teacher candidates focused on the difficulties they experienced in expressing themselves. This highlights that speech anxiety is a key factor limiting communication skills in both experienced teachers and teacher candidates, and emphasizes the importance of reducing anxiety for effective teaching processes.

When comparing responses to reduce or reduce speaking anxiety, both experienced and prospective social studies teachers focused on topic preparation. This indicates that preparation is a central strategy for reducing anxiety, and that planned study and pre-speaking preparation are effective coping mechanisms for both experienced teachers and teacher candidates.

There are numerous studies on speaking anxiety, both domestically and internationally. What distinguishes this study from existing research is the lack of a detailed comparison of the speaking anxiety of social studies teachers and prospective social studies teachers. This study aims to address this deficiency. Below, studies on speaking anxiety and their similarities and differences with our study are presented.

Balamir (2009), in a study examining the relationship between the reasons for students’ speaking anxiety and students’ foreign language speaking and language proficiency levels, revealed that students have a medium level of foreign language speaking anxiety. It was also concluded that language proficiency levels do not play a significant role in foreign language speaking anxiety levels. Furthermore, Balademir determined that teaching methods and evaluation methods, personal reasons, and fear of negative evaluation are among the reasons that cause anxiety. In this study, it was revealed that the reasons for speaking anxiety include the attitudes of the other party, fear of being judged, fear of failure, being embarrassed (being ridiculed and humiliated), disturbing looks from listeners, and fear of making mistakes. Balamir (2009) study and this study are similar in the results they present.

In a 2014 study, Bas aimed to determine the factors that cause foreign-language learning anxiety in high school students. As a result, it was concluded that the factors that cause foreign language learning anxiety in high school students were grouped into seven themes: namely speaking activities, listening activities, teaching methods and techniques, fear of making mistakes, learning environment, teacher attitudes, and exams. When the results of this study are examined, it is seen that the factors that cause speaking anxiety are: fear of not being able to express oneself, fear of being the center of attention, the thought of feeling inadequate, attitudes of the other party, fear of being judged, fear of failure, fear of being disgraced, and fear of making mistakes. The findings of the study are consistent with those of Bas (2014).

Iscan and Karagoz (2016), who aimed to determine the speaking anxiety levels of undergraduate students in the Turkish language teaching department, stated that students’ inadequate field knowledge, lack of self-confidence, and reluctance to speak in front of a crowd contributed to this result. When the results of this study are examined, it is seen that the factors causing speaking anxiety are: fear of not being able to express oneself, fear of being the center of attention, the thought of feeling inadequate, attitudes of the other party, fear of being judged, fear of failure, fear of being disgraced, and fear of making mistakes. The results of this study are consistent with those of Iscan and Karagoz (2016). Sutarsyah (2017) aimed to reveal whether speaking anxiety affects students’ speaking performance and the effects of anxiety levels on speaking performance in his study titled “Analysis of students’ speaking anxiety and its effect on speaking performance.” At the end of the study, it was revealed that speaking anxiety levels affect students’ speaking performance. The results of this study are similar to Sutarsyah (2017) study.

Gunduz and Demir (2022), in their study aiming to determine the opinions of middle school students experiencing speech anxiety, determined that these anxieties of students experiencing speech anxiety are generally due to their own faults and listener-related stimuli. The present findings are similar to those obtained by Gunduz and Demir (2022). In this study, it is seen that the reasons for speech anxiety are; fear of not being able to express oneself, fear of being the center of attention, the thought of feeling inadequate, the attitudes of the other party, fear of being judged, fear of failure, being embarrassed (being ridiculed, humiliated), excitement, the crowd being crowded, the thought of not being able to meet expectations, the disturbing looks of the listeners, fear of making mistakes and shyness.

Based on the findings of our study, the following recommendations have been made:

- Practical courses focusing on communication and oral expression skills can be increased in teacher training programs.
- Psycho-educational interventions addressing the affective dimension of speech anxiety can be included.
- Practices aimed at strengthening prospective teachers’ self-efficacy perception can be developed.
- Planned speaking and topic preparation skills can be systematically taught.
- Future research can examine speech anxiety in terms of variables such as gender, length of professional experience, grade level, and personality traits, and mixed-methods studies using both qualitative and quantitative methods can be conducted.

## Data Availability

The original contributions presented in the study are included in the article/supplementary material; further inquiries can be directed to the corresponding author.
